# Fabrication and Electrical Characterization of MISFET Structures with SiO_2_/HfO_x_N_y_ Gate Dielectric Stack

**DOI:** 10.3390/nano16201294

**Published:** 2026-10-13

**Authors:** Piotr Firek, Piotr Wysokiński

**Affiliations:** Institute of Microelectronics and Optoelectronics, Warsaw University of Technology, Koszykowa 75, 00-662 Warsaw, Poland; pwysokin@gmail.com

**Keywords:** magnetron sputtering, HfO_X_N_Y_, MISFET, ISFET, electronics properties, high-k dielectrics, thin film

## Abstract

In this research, the fabrication process of MISFET (Metal–Insulator–Semiconductor Field-Effect Transistor) structures with a SiO_2_/HfO_x_N_y_ stack as a gate dielectric was developed, providing a viable device platform for future ion-sensitive and biosensing transducer integration. Hafnium Oxide-Nitride films were deposited using reactive magnetron sputtering in an O_2_/N_2_/Ar gas mixture. The thickness of HfO_x_N_y_ films was measured using spectroscopic ellipsometry. To evaluate the electrical properties, MIS (metal–insulator–semiconductor) structures were formed by Al metallization. Then layers were electrically characterized using I-V and C-V measurements. This allowed the extraction of key electrical parameters such as flat-band voltage V_FB_, dielectric constant K_i_, interface state trap density D_it_, and effective charge Q_eff_. Subsequently, deposited HfO_x_N_y_ layers were annealed in a post-deposition annealing (PDA) process (40 min, 400 °C, 100% N_2_) followed by a second electrical characterization. Finally, MISFET structures were developed, incorporating the HfO_x_N_y_ gate dielectric layer previously characterized. The transfer and output current–voltage (I-V) characteristics of the produced transistors are presented and discussed.

## 1. Introduction

Ion-Sensitive Field-Effect Transistor (ISFET) micro-sensors have been widely used for applications in pH monitoring, analytical chemistry, and biomedical sensing [1]. Due to its unique architecture, the ISFET can be used as an excellent platform for the investigation of various materials for novel sensitive membranes. Instead of the commonly used stacked gate structure of SiO_2_/Si_3_N_4_, new dielectric materials to enhance device sensitivity and long-term stability (e.g., DLC, Al_2_O_3_, Ta_2_O_5_, BaTiO_3_) can be applied [2,3,4,5].

Among these candidates, hafnium oxynitride stands out as a promising high-k dielectric. It is characterized by a high crystallization temperature, exceptional chemical resistance, and superior interface quality when integrated with semiconductor substrates [6]. These attributes have led to its extensive use in various advanced electronic applications [7,8,9]. Recent advancements in dielectric engineering have further underscored the crucial role of interface control and defect passivation in high-performance field-effect transistors [10]. Furthermore, incorporating an ultrathin thermal SiO_2_ buffer beneath the high-k layer prevents uncontrolled intermixing at the silicon interface, mitigating high interface state densities while maintaining the capacitance benefits of the oxynitride.

The primary advantage of HfO_x_N_y_ over pure Hafnium Oxide HfO_2_ lies in the role of nitrogen within the atomic network. The incorporation of nitrogen effectively suppresses grain growth and elevates the crystallization temperature [11]. This allows the film to remain in an amorphous state during subsequent high-temperature processing steps [12]. Amorphous layers are highly desirable as they exhibit significantly lower leakage currents and superior dielectric uniformity compared to polycrystalline films, where grain boundaries act as preferential conduction paths for charge carriers [13].

Although nitrogen incorporation may slightly reduce the bandgap of hafnium-based dielectrics compared to pure HfO_2_, it can improve the electrical and interface quality of the gate stack. Nitrogen atoms incorporated into the oxide network tend to interact with oxygen vacancy sites, repelling vacancy-related gap states toward band edges and thus mitigating charge trapping associated with these defects; this reduction in charge trapping and leakage current is commonly observed in high-k oxides with nitrogen incorporation [14]. Additionally, experimental studies on HfO_x_N_y_ films show that increasing nitrogen content lowers the optical bandgap while simultaneously improving interface stability and reducing leakage and hysteresis in MOS structures, consistent with the beneficial role of nitrogen in passivating defect states and enhancing dielectric reliability [15].

The electrical parameters of HfO_x_N_y_ layers are highly dependent on the fabrication method, as the material is inherently non-stoichiometric. Research indicates that optimal properties are typically achieved at a moderate nitrogen content in the range of ~8–16 at.% [16]. Under these conditions, HfO_x_N_y_ films exhibit a reduced optical bandgap compared to pure HfO_2_, a refractive index on the order of ~2.0 [17], and high-k behavior with dielectric constant values in the range ~15–25 depending on deposition and processing conditions [18].

The deposition of HfO_x_N_y_ thin films is most commonly performed using Physical Vapor Deposition (PVD) techniques. The literature describes several successful approaches, including reactive magnetron sputtering in an O_2_/N_2_/Ar atmosphere [19], reactive sputtering in a pure N_2_ ambient followed by post-deposition annealing (PDA) [20], and Atomic Layer Deposition (ALD) [21]. In this work, we focus on the fabrication technology and electrical optimization of MISFET structures utilizing a SiO_2_/HfO_x_N_y_ gate stack. The primary objective is to establish a reliable manufacturing framework and stabilize the device threshold voltage, facilitating the subsequent use of this dielectric architecture as a transducer platform for future chemical and biological sensing applications.

## 2. Experimental Details

**Figure 1:**
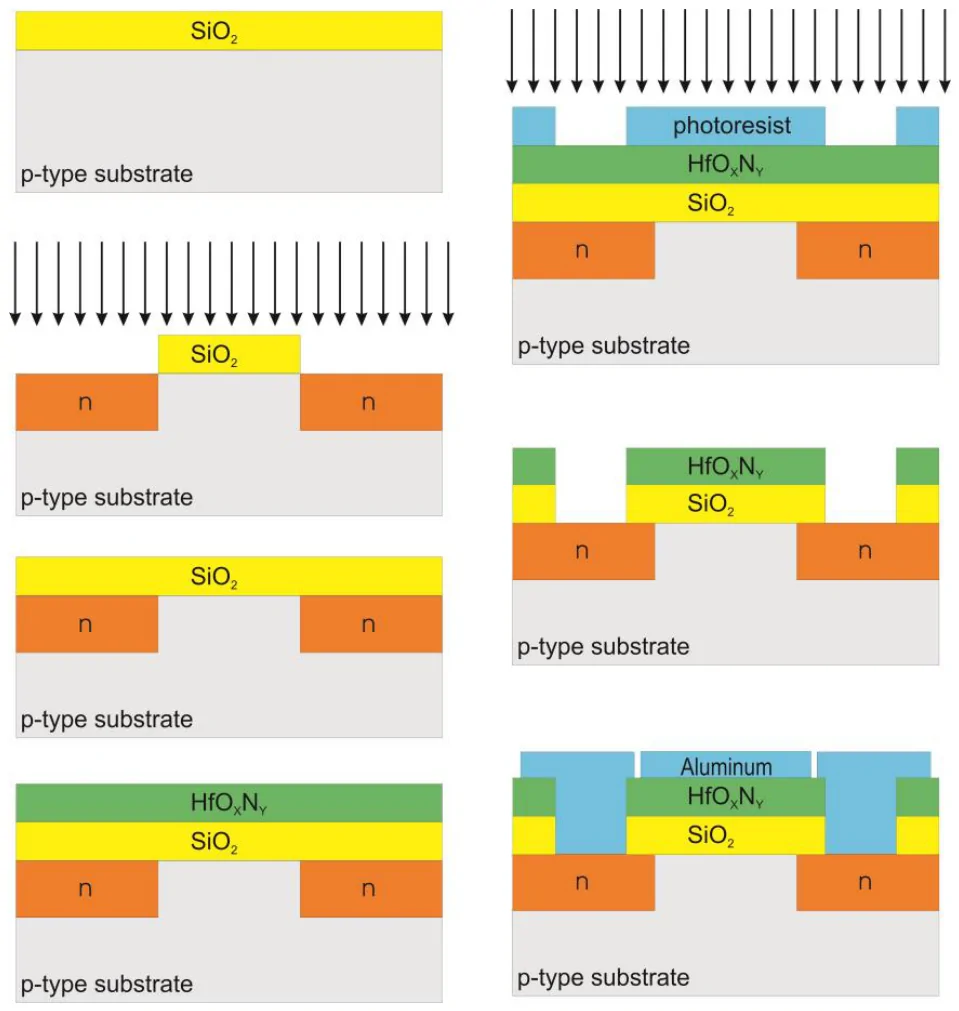
Schematic process flow for the fabrication of transistor structures with a double SiO_2_/HfO_x_N_y_ gate stack. Dimensions and thicknesses: initial field oxide = 440 nm; active gate stack = 20 nm thermal SiO_2_ buffer + 50 nm sputtered HfO_x_N_y_ (total physical gate thickness t_ox_ = 70 nm); transistor channel length L = 15 µm, channel width W = 600 µm (aspect ratio W/L = 40); test MIS capacitor contact diameter ϕ = 1 mm (A~7.85 × 10^−3^ cm^2^).

The fabrication process of transistor structures employing a double SiO_2_/HfO_x_N_y_ gate dielectric is illustrated in Figure 1. The first step of the fabrication process of MIS/ISFET structures is thermal oxidation in order to obtain a field oxide of about 440 nm. A p-type <100> silicon substrate with a resistivity of 1–10 Ωcm was used. After the first level of photolithography, the drain and source regions were opened and doped with phosphorus. In the next step, a 20 nm thick SiO_2_ film was produced by silicon thermal oxidation, and then a 50 nm thick HfO_x_N_y_ film was deposited using an Oxford Instruments Plasmalab 400 magnetron and Hf target, yielding a total gate dielectric stack thickness of 70 nm. It should be noted that the initial 440 nm thick field oxide was completely etched away in the active gate area down to the silicon substrate prior to the growth of the high-quality 20 nm thick gate thermal oxide.

Through process optimization using an L_9_(3^4^) orthogonal table, the influence of deposition process parameters on the electronic properties of obtained layers was examined. The deposition process was conducted in a mixed oxygen and nitrogen atmosphere. Based on the previous results, the most promising material for the ISFET applications can be obtained under the following process conditions: power P = 800 W, pressure p = 3 mTorr or 6 mTorr, Ar flow = 50 sccm, and N_2_/O_2_ gas ratio = 0.5 [22].

Another level of photolithography defines a pattern in the photoresist mask for the plasma reactive ion etching process (RIE). Etching was carried out using an Oxford Instruments Plasmalab system. The etching process parameters for HfO_x_N_y_ are summarized in Table 1 [23]. In the last step, windows for metal evaporation were opened and aluminum was deposited. The photolithography masks used in the last process allow the manufacturing of two types of transistors on a single wafer: MISFET structures with an aluminum gate and ISFET structures without a gate electrode.

The resulting MISFET devices were fabricated with a nominal channel length of L = 15 µm and a channel width of W = 600 µm, yielding an aspect ratio of W/L = 40. For simultaneous dielectric monitoring and capacitance–voltage (C-V) characterization, circular MIS test capacitors with a contact diameter of ϕ = 1 mm (gate area A~7.85 × 10^−3^ cm^2^) were fabricated. 

The current–voltage (I-V) characteristics of SiO_2_/HfO_x_N_y_-based MISFET structures were measured with Keithley SMU 236/237/238.

The high-frequency (100 kHz) capacitance–voltage (C-V) characteristics of the fabricated Al/HfO_x_N_y_/SiO_2_/p-Si MIS capacitor structures were measured at room temperature. To analyze hysteresis phenomena and mobile/trapped charge dynamics, C-V sweeps were performed from inversion to accumulation and back to inversion. The key electrophysical parameters of the gate dielectric stack were extracted from the measured high-frequency C-V curves as follows [24,25,26].

The equivalent gate stack capacitance and the relative dielectric permittivity of the high-k layer were determined from the maximum capacitance in strong accumulation (C_max_) according to

$$\varepsilon_{r}=\frac{C_{\max}\cdot X_{i}}{\varepsilon_{0}\cdot A}$$

where ε_0_ is the vacuum permittivity, A is the gate area (7.85 × 10^−3^ cm^2^), and X_i_ is the physical dielectric thickness.

The flat-band capacitance (C_FB_) was calculated using the standard depletion approximation, taking into account the silicon substrate doping concentration (N_B_) and thermal voltage (kT/q). The flat-band voltage (V_FB_) was then directly extracted from the experimental C-V curves at C = C_FB_.

$$C_{\mathrm{FB}}=\frac{1}{\frac{1}{C_{\max}}+\frac{1}{A\cdot q}\cdot \sqrt{\frac{k\cdot T}{\varepsilon_{s}\cdot N_{B}}}}$$

The hysteresis loop width (ΔV_H_) was quantified as the voltage shift between the forward and reverse sweeps in the vicinity of the flat-band condition.

The interface trap density (D_it_) was evaluated using the simplified high-frequency Terman method. The midgap capacitance (C_MB_) was calculated via the depletion approximation, and the experimental voltage stretch-out (ΔU_G_) between predetermined surface potential points around midgap (ϕ_S−1_ to ϕ_S+1_) was compared with the theoretical ideal voltage shift (ΔU_G0_), yielding

$$D_{\mathrm{it}}=\frac{C_{\max}\cdot \left(\Delta U_{G}-\Delta U_{G(i)}\right)}{2\cdot k\cdot q\cdot T\cdot A}$$

The effective charge density in the dielectric stack (Q_eff/q_) was calculated from the flat-band voltage shift according to

$$Q_{\mathrm{eff}}=\frac{\varepsilon_{i}\cdot \left(\phi_{\mathrm{ms}}-U_{\mathrm{FB}}\right)}{X_{i}}$$

where ϕ_ms_ is the metal–semiconductor work function difference for the p-type silicon substrate.

## 3. Results and Discussion

To evaluate the impact of the pattern transfer process on the structural integrity and electrical properties of the gate stack, three distinct reactive ion etching (RIE) conditions were investigated, as detailed in Table 1. Etching of high-k HfO_x_N_y_ layers requires a precise balance between chemical etching (driven by reactive fluorine radicals from CF_4_) and physical sputtering (induced by heavy Ar^+^ ion bombardment). Sample 1 was etched in a pure CF_4_ atmosphere at an RF power of 250 W, relying predominantly on chemical reactions. Although this regime operates with a limited physical component—as the RIE reactor configuration inherently enforces a physical sputtering aspect even in the absence of argon- it mitigates severe ion bombardment, though it may risk undercutting or leave non-volatile etch byproducts at the gate periphery. In contrast, Samples 2 and 3 utilized a 1:1 CF_4_/Ar gas mixture, where Ar^+^ ion sputtering assists in breaking Hf-O and Hf-N bonds while facilitating the desorption of etch byproducts to yield sharper sidewall profiles. Furthermore, the results highlight the critical role of plasma power: reducing the RF power to 200 W (for Sample 3, down from 250 W for Sample 2) decreases the energy of bombarding ions, thereby mitigating plasma-induced damage. Such plasma-induced charges and edge defect states directly influence the initial threshold voltage Vth shifts and electrical performance prior to the post-deposition annealing (PDA) process.

**Figure 2:**
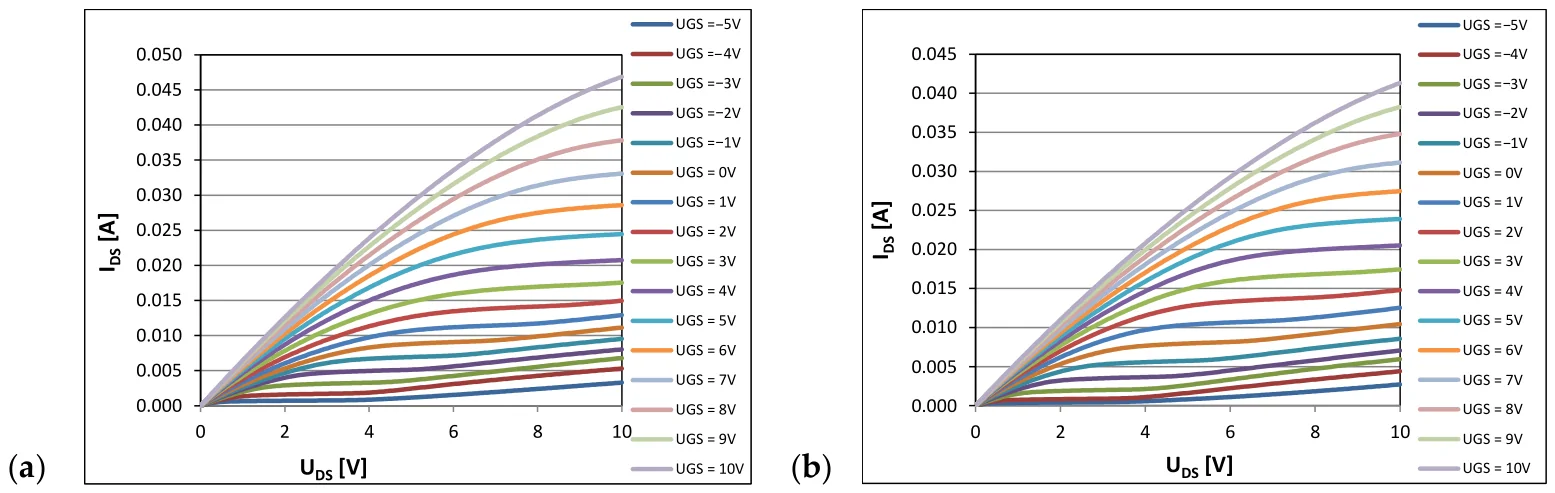

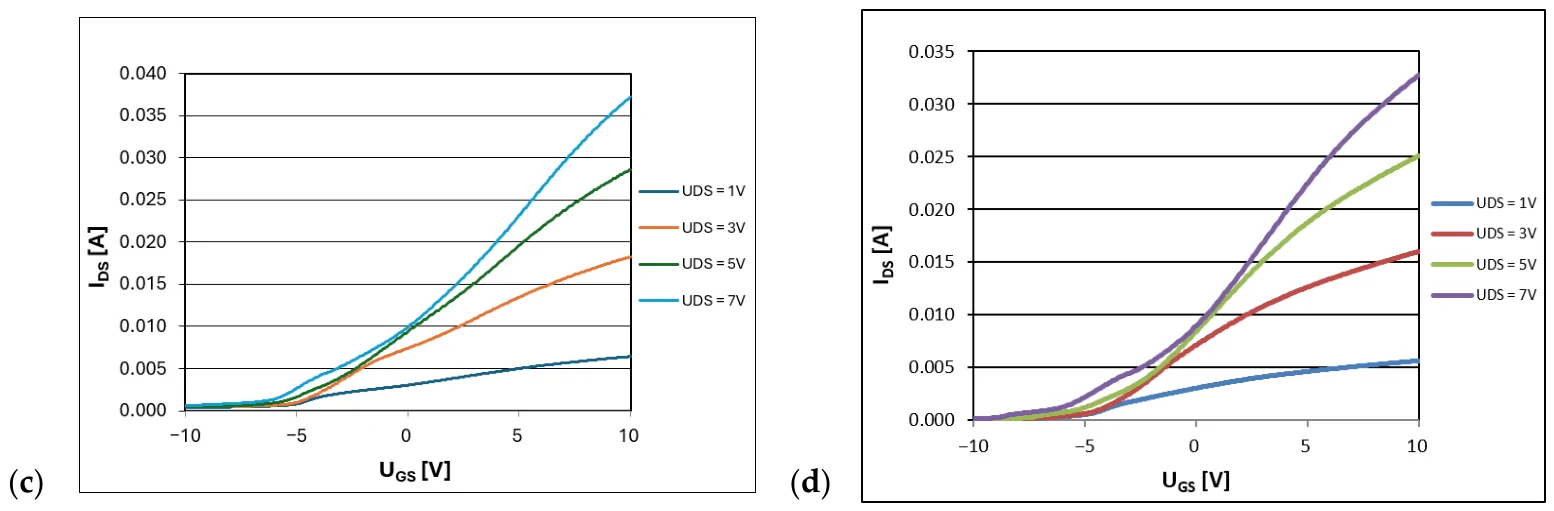
Output characteristics of MISFET transistor—Sample 1: (**a**) before PDA and (**b**) after PDA. Transient characteristics of MISFET transistor: (**c**) before PDA and (**d**) after PDA.

**Figure 3:**
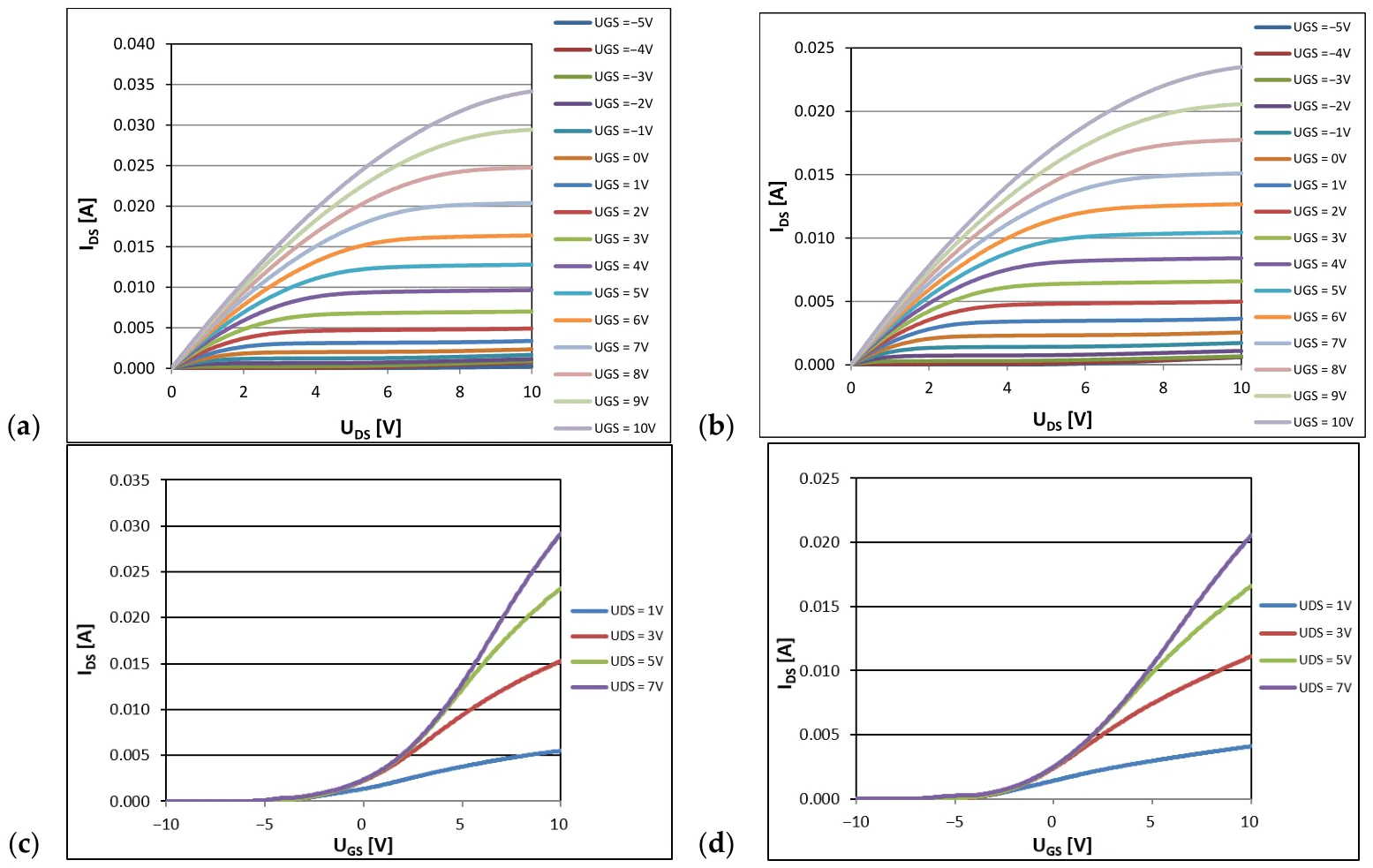
Output characteristics of MISFET transistor—Sample 2: (**a**) before PDA and (**b**) after PDA. Transient characteristics of MISFET transistor: (**c**) before PDA and (**d**) after PDA.

**Figure 4:**
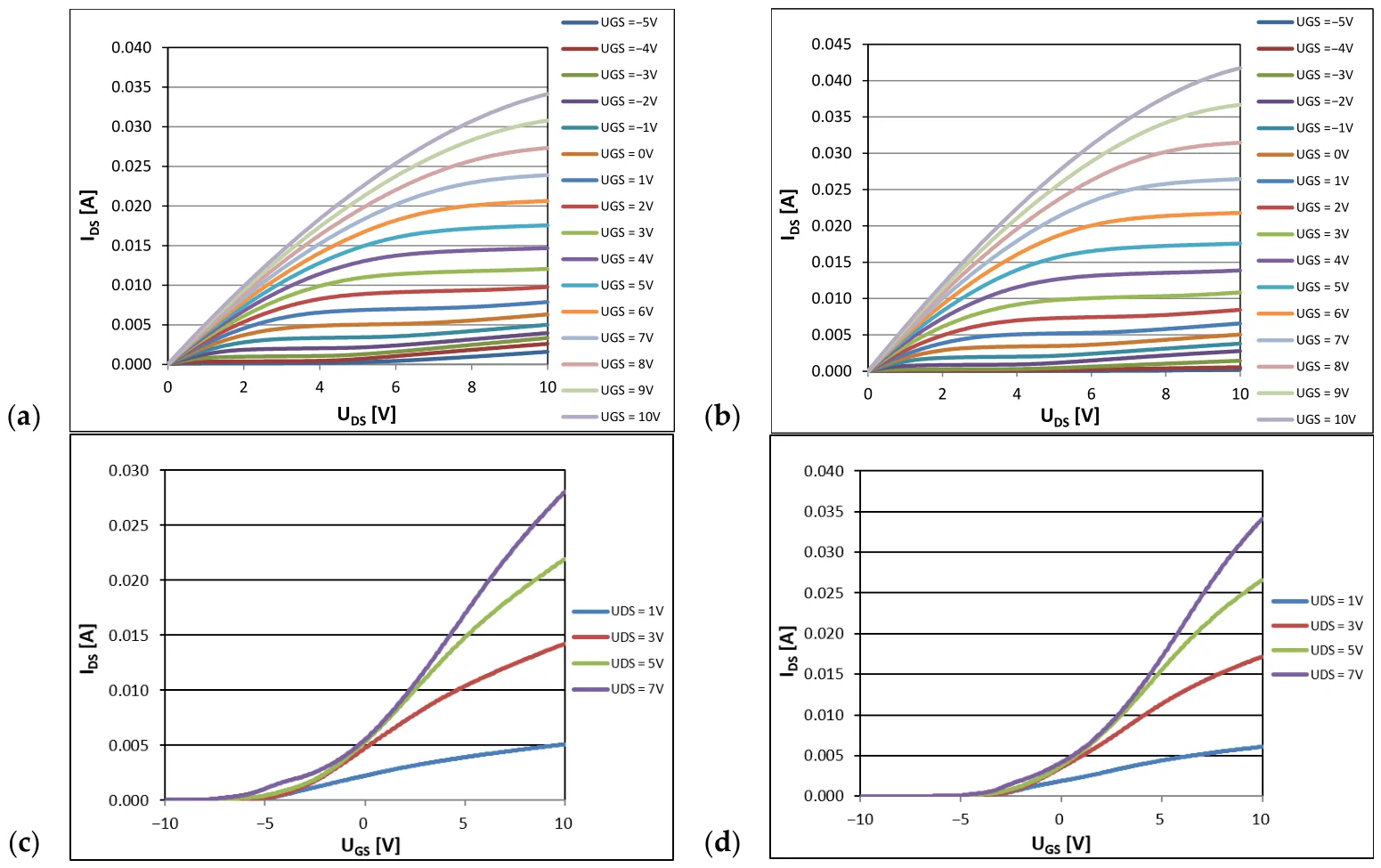
Output characteristics of MISFET transistor—Sample 3: (**a**) before PDA and (**b**) after PDA. Transient characteristics of MISFET transistor: (**c**) before PDA and (**d**) after PDA.

Typical transfer and output characteristics of samples from Table 1 are presented in Figure 2, Figure 3, and Figure 4, respectively. In the case of MISFETs, the threshold voltage was within the range of −11.53 V to −4.81 V. The values are too high and unacceptable for standard MOSFET technology. For future use of the stack as a membrane in ISFET structures, the Vth value should also be improved. Thermal annealing was applied to decrease the threshold voltage (time t = 40 min, temperature of 400 °C, and N_2_ atmosphere). Following the annealing process, the threshold voltage shifted to a range of −5.4 V to −4.72 V, representing a significant improvement in device performance. This substantial reduction in threshold voltage demonstrates the effectiveness of thermal annealing in improving the electrical stability and performance of the HfO_x_N_y_ gate stack. Simultaneously, the gate dielectric insulation integrity was monitored. Across all measured transfer characteristics (V_GS_ swept from −10 V to +10 V), the gate leakage current (I_G_) remained at very low levels, consistently below 10^−8^ A (<10 nA), confirming that drain current modulation was not affected by parasitic gate conduction. This behavior is fully consistent with the low leakage current levels (0.1–1.0 nA) observed on the dedicated test MIS capacitor structures [22].

The observed reduction in the threshold voltage following the PDA process can be attributed to the reorganization of the atomic structure and the passivation of defect states within the HfO_x_Ny matrix. In the as-deposited state, high-k dielectrics often exhibit a significant density of fixed positive charges and oxygen vacancies, which act as trap centers and shift the Vth toward more negative values in p-channel devices or excessively high values in n-channel structures [27]. Thermal treatment in a nitrogen ambient facilitates the relaxation of the HfO_x_N_y_ network and reduces the effective oxide charge at the Si/SiO_2_/HfO_x_N_y_ interfaces.

Furthermore, the nitrogen atoms incorporated into the film help to passivate dangling bonds and oxygen vacancies, leading to a more stable and lower interface trap density, which directly correlates with the observed improvement in Vth stability and its shift toward a more acceptable operational range [14]. Indeed, recent investigations into advanced gate dielectrics have emphasized that the chemical coordination, local bonding environment, and defect state modulation play a decisive role in governing charge transport and dielectric reliability [28]. These mechanisms are quantitatively corroborated by the electrophysical parameters extracted from MIS capacitor structures, summarized in Table 2. Following the PDA treatment, the dielectric permittivity K_i_ increases from ~15.7 to ~18.3, while the flat-band voltage V_FB_ exhibits a notable shift from −6.8 V to −2.9 V. Concurrently, the effective positive charge density (Q_eff/q_) decreases from 1.1 × 10^12^ cm^−2^ to 0.7 × 10^12^ cm^−2^, and the midgap interface trap density (D_it_) drops substantially from 6.6 × 10^12^ eV^−1^cm^−2^ to 2.9 × 10^12^ eV^−1^cm^−2^ [22]. Furthermore, the marked reduction in the C-V hysteresis width (ΔV_H_ from ~0.65 V down to ~0.18 V) indicates the effective suppression of border traps and mobile charge redistribution near the interface. This collective decline in bulk oxide charge and interface states provides clear electrophysical validation for the observed stabilization and favorable threshold voltage shift (Vth) in the fabricated MISFET devices.

## 4. Conclusions

In conclusion, this study successfully demonstrated the fabrication and characterization of MISFET structures utilizing a SiO_2_/HfO_x_N_y_ gate dielectric stack. The electrical characteristics of the fabricated transistors showed excellent repeatability, confirming the stability of the deposition process. While the initial devices exhibited high threshold voltages, the application of post-deposition annealing (PDA) at 400 °C in a nitrogen ambient proved to be an effective strategy for Vth stabilization and reduction. This improvement was supported by MIS capacitor data, which revealed a significant decrease in both effective oxide charge Q_eff_ and interface trap density D_it_.

However, to reach the implementation stage for commercial applications, further optimization is required. Future work will focus on refining the selective etching process for hafnium oxynitride to ensure precise device patterning. Additionally, the improved HfO_x_N_y_ stack will be integrated as a sensing membrane in ISFET structures, where its high-k properties and enhanced thermal stability are expected to improve sensitivity and chemical robustness in biological and gas sensing applications.

## Figures and Tables

**Table 1:** HfO_x_N_y_ etching process parameters.

	Power	Pressure	Flow CF_4_/Ar	Time
Sample	[W]	[mTorr]	[sccm/sccm]	[min]
1	250	30	40/0	2:30
2	250	30	20/20	2:30
3	200	30	20/20	2:30

**Table 2:** Key electrophysical parameters of MIS capacitor structures with HfO_x_N_y_ gate dielectric before and after post-deposition annealing, extracted from high-frequency C-V measurements (data adapted from the baseline dielectric optimization [22]).

Parameter	As-Deposited (Before PDA)	After PDA (400 °C, N2)	Physical Effect/Impact on MISFET
Dielectric constant K_i_	~15.7	~18.3	Network densification, increased gate capacitance
Flat-band voltage V_FB_ [V]	−6.8	−2.9	Significant positive shift toward ideal flat-band
Hysteresis loop width ΔV_H_ [V]	~0.65	~0.18	Elimination of slow border traps/charge trapping
Effective oxide charge Q_eff/q_ [10^12^ cm^−2^]	1.1	0.7	Reduction in fixed positive charges and oxygen vacancies
Interface trap density D_it_ [10^12^ eV^−1^ cm^−2^]	6.6	2.9	Passivation of Si/dielectric interface states

## Data Availability

The raw data supporting the conclusions of this article will be made available by the authors on request.
